# The efficacy of iodine-125 permanent brachytherapy versus intensity-modulated radiation for inoperable salivary gland malignancies: study protocol of a randomised controlled trial

**DOI:** 10.1186/s12885-016-2248-7

**Published:** 2016-03-07

**Authors:** Shu-Ming Liu, Hai-Bo Wang, Yan Sun, Yan Shi, Jie Zhang, Ming-Wei Huang, Lei Zheng, Xiao-Ming Lv, Bao-Min Zheng, Kathleen H. Reilly, Xiao-Yan Yan, Ping Ji, Yang-feng Wu, Jian-Guo Zhang

**Affiliations:** Department of Oral and Maxillofacial Surgery, Peking University School and Hospital of Stomatology, 22 Zhongguancun South St, Haidian Dist Beijing, 100081 PR China; Peking University Clinical Research Institute, Xueyuan Rd 38#, Haidian Dist Beijing, 100191 PR China; Department of radiotherapy, Peking University Cancer Hospital & Institute, 52 Fucheng Road, Haidian Dist Beijing, 100142 PR China; Independent Consultant, New York, NY USA

**Keywords:** I-125 permanent brachytherapy, Intensity-modulated radiation therapy, Inoperable salivary gland malignancy, Local control rate, Quality of life

## Abstract

**Background:**

Radiation therapy is the method of choice for subjects with inoperable salivary gland malignancies. I-125 brachytherapy, delivering a high radiation dose to a tumor but sparing surrounding normal tissues, is supposed to be ideal modality for the treatment of salivary gland malignancies. We designed a randomised controlled clinical trial to compare the efficacy of I-125 permanent brachytherapy (PBT) versus intensity-modulated radiation therapy (IMRT) for inoperable salivary gland malignancies.

**Methods/Design:**

In this study, inclusion criteria are subjects with inoperable salivary gland malignancies, aged 18–80 years, have provided informed consent, with at least one measurable tumor focus, be able to survive ≥3 months, Karnofsky performance status ≥60, have adequate hematopoietic function of bone marrow, have normal liver and kidney function, and are willing to prevent pregnancy.

Exclusion criteria include a history of radiation or chemotherapy, a history of other malignant tumors in the past 5 years, receiving other effective treatments, participating in other clinical trials, with circulatory metastasis, cognitive impairment, severe cardiovascular and cerebrovascular diseases, acute infection, uncontrolled systemic disease, history of interstitial lungdisease, and being pregnant or breast feeding.

The study will be conducted as a clinical, prospective, randomised controlled trial with balanced randomisation (1:1). The planned sample size is 90 subjects. Subjects with inoperable salivary gland malignancies are randomised to receive either I-125 PBT or IMRT, with stratification by tumor size and neck lymph node metastasis. Participants in both groups will be followed up at 2, 4, 6, 9, 12, 15, 18, 21 and 24 months after randomization. The primary outcome is local control rate of the primary site (based on imaging findings and clinical examination, RECIST criteria) in 1 year. Secondary outcomes are progression-free survival, overall survival, quality of life (QOL) measured with the European Organization for Research and Treatment of Cancer QOL Questionnaire (EORTC QLQ-C30 and QLQ-H&N35) of Chinese version, and safety of treatment. Chi-squared test is used to compare the local control rates in both groups. The survival curves are estimated by the Kaplan-Meier method, and log-rank test is used to test the significant difference.

**Discussion:**

Only few observational studies have investigated the effect of I-125 PBT on inoperable salivary gland malignancies. To our knowledge, this is the first randomised controlled trial to investigate the efficacy of I-125 PBT for subjects with inoperable salivary gland malignancies, and will add to the knowledge base for the treatment of these subjects.

**Trial registration:**

The study is registered to Clinical Trials.gov (NCT02048254) on Jan 29, 2014.

## Background

Primary malignant tumors of the major and minor salivary glands are relatively rare entities, accounting for only about 3–5 % of all head and neck malignancies. They also contain a diverse group of histologies, with disparate characteristics in terms of aggressiveness and patterns of spread [1]. Surgery has historically been the mainstay of treatment for salivary gland malignancies. Subjects with low-grade tumors are usually treated with surgery alone if complete excision can be achieved. Radiotherapy, as a postoperative adjunct to surgery, has traditionally been reserved for subjects with microscopically high-grade tumors, positive margins or nerve invasion. Although carcinomas originating from the salivary glands were previously thought to be radioresistant, the role of adjunct radiotherapy in therapy has become well recognized [2, 3]. However, the therapy for inoperable malignant salivary has been extremely challenging in clinical practice: some subjects are either not candidates for definitive resection or undergo limited procedures leaving behind gross residual disease. Typically, these subjects are deemed inoperable because of technical issues related to the extensiveness or location of the primary tumor. Another subset of inoperable subjects present with medical comorbidities that places them at unacceptably high risk for perioperative complications. Lastly, some subjects refuse surgical therapy out of personal preference. For whatever reason, these inoperable subjects have all traditionally been offered definitive radiation therapy as an alternative to surgery. Unfortunately, the reported results following low linear energy transfer (LET) irradiation are poor, with overall local control rates average below 30 % [4–10]. In a multicenter randomised controlled study conducted by the American Radiation Therapy Oncology (RTOG) and the UK Medical Research Council (MRC), the 2-year local control rate (LCR) of unresectable salivary gland cancers with conventional external radiotherapy was only 17 % [10].

The dose response of biological systems is influenced by the LET of ionizing radiation. In general, relative radiobiological effectiveness (RBE) increases with LET. A number of studies have been carried out to investigate the effectiveness of high-LET RT such as fast neutron RT. Batterman et al. described an elevated RBE for fast neutrons in the treatment of lung metastases of malignant salivary gland tumors. In this study, the highest RBE values up to 8 were found for ACC [11]. The randomised controlled study conducted by RTOG and MRC showed that the 2-year local control rate was 67 % for fast neutron radiotherapy compared with 17 % for conventional photon radiation [10]. However, the indication of fast neutron radiotherapy has been strictly limited because of its unacceptable damage to the surrounding normal tissues [12, 13]. In heavy particles radiotherapy, carbon ions have similar radiobiologic properties as neutrons, and higher RBE values can be expected for salivary gland tumors. Compared with neutron RT, carbon ions additionally provide physical selectivity due to an inverse dose profile. Thus, carbon ions was considered to have potentially greater clinical value [14]. In Germany, intensity-modulated radiation therapy (IMRT) combined with carbon ions radiotherapy was recommended as the standard treatment for inoperable salivary gland malignancies [15]. But carbon ions radiotherapy was expensive and unavailable, which obviously limits its application.

Brachytherapy is an important modality in the treatment of human malignancy with ionizing radiation. Where applicable, it may be the method of choice for the following reasons [16, 17]. First, the localized dose distribution enhances the ratio of tumor dose to surrounding normal tissue dose. Second, the reduction of oxygen enhancement ratio and dose rate may partially circumvent the radioresistance of hypoxic tumor cells. Permanent implants of I-125 sources in focus sites have been widely used, especially for prostatic cancer [18]. Because LET increases with decreasing photon energy, I-125 source with low energy photons(average 28 keV) has higher RBE values (approximately 1.4) [19]. In addition, the low photon energy also provides more sparing for adjacent normal tissue and easy resolution of the problem of protecting medical staff from radiation exposure. Some studies have shown that I-125 permanent brachytherapy (PBT) may have potential advantages in local control of salivary gland malignancies and in minimizing radiobiological damage to normal adjacent tissues [20–24]. I-125 has a long half-life of 60 days and may be ineffective in eradicating tumors with fast growth kinetics [25–27]. The clinical efficacy of I-125 in prostatic cancer may be due to the relative slow proliferative rate of this disease [25–27]. Like prostatic cancer, many of salivary gland malignancies are characteristically slowly proliferating tumours with long natural histories. Therefore, I-125 is supposed to be ideal modality for the treatment of salivary gland malignancies, as suggested by other nonrandomised clinical trials. In order to obtain more credible evidence, we launch a Phase III, randomised controlled trial to compare the efficacy of I-125 PBT versus IMRT for inoperable salivary gland malignancies.

## Methods and design

### Trial design and setting

The study is conducted as a single center, prospective, randomised controlled trial with balanced randomisation (1:1) for subjects who have inoperable malignant salivary gland tumors are randomised to receive either I-125 PBT or IMRT. Subjects are stratified by tumor size (≤4 cm vs. >4 cm) and neck lymph node metastasis (yes vs. no). Subjects are recruited continuously by oncologists at Department of Oral and Maxillofacial Surgery, Peking University School and Hospital of Stomatology which is the only department to treating salivary gland malignancies by I-125 PBT in China.

### Ethical approval

**Fig. 1:**
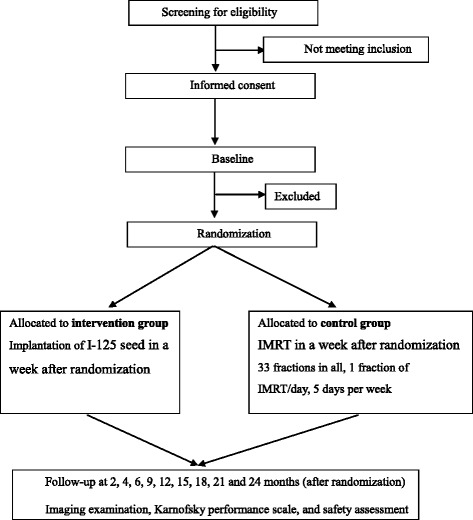
Study flow chart

The protocol and informed consent form have been reviewed and approved by the Institutional Review Boards of the Peking University Health Science Center in Beijing, China, and registered at www.clinicaltrials.gov (NCT02048254). Figure 1 illustrates the flow diagram of the study for both the intervention and control groups.

### Participants

Ninety subjects will be recruited for the study and divided into an intervention group (I-125 PBT) and a control group (IMRT).

### Inclusion criteria

In the study, inclusion criteria are as follows: (1) willing to participate and sign informed consent; (2) aged 18–80 years; (3) malignancies originating from major or minor salivary glands based on pathological and/or cytological diagnosis, including primary or recurrent tumors; (4) with inoperable tumors, including unresectable primary locally advanced tumors; unresectable recurrent tumors; and unable to undergo surgery due to other medical comorbidities or refusal of surgery out of personal preference, and being T3/T4 T-stage; (5) with at least one measurable tumor focus based on RECIST criteria and imaging diagnosis completed in 30 days before enrollment; (6) expected survival time ≥3 months; (7) Karnofsky performance status ≥60; (8) adequate hematopoietic function of bone marrow in previous 7 days: hemoglobin ≥9 g/dL, white blood cell count ≥3.0 × 10^9^/L, neutrophils count ≥1.5 × 10^9^/L, platelet count ≥100 × 10^9^/L; (9) normal liver and kidney function in previous 14 days: total bilirubin in serum ≤1.5 times the upper limit of normal (ULN), alanine transaminase and aspartate transaminase ≤3 times ULN, creatinine ≤1.5 times ULN; (10) willing to take measures to prevent pregnancy.

### Exclusion criteria

Exclusion criteria include: (1) with a history of radiation treatment on head and neck; (2) with a history of other malignant tumors in past 5 years, except for healed skin basal cell carcinoma and cervical carcinoma in situ; (3) with a history of tumor chemotherapy; (4) receiving other effective treatments; (5) having participated in other clinical trials in 4 weeks before enrollment; (6) with circulatory metastasis; (7) with the histology subtype of squamous cell carcinoma; (8) without measurable tumor focus, such as diffuse infiltrative carcinoma; (9) cognitive impairment due to neursis or psychosis; (10) cardiovascular and cerebrovascular diseases with clinical significance, such as heart failure in NYHA III/IV, uncontrolled coronary heart disease, myocardiopathy, uncontrolled arrhythmia, uncontrolled hypertension, history of myocardial infarction or cerebral infarction in the past half year; (11) severe clinical infection in 14 days before randomization including active pulmonary tuberculosis; (12) human immunodeficiency virus infection, active hepatitis B or hepatitis C; (13) uncontrolled systemic disease, such as diabetes mellitus; (14) with the history of interstitial lung disease, such as interstitial pneumonia, pulmonary fibrosis, or diagnosed as interstitial lung disease by chest X-ray/CT image; (15) being pregnant or breast feeding.

### Withdrawal of individual subjects

Subjects can withdraw from the study at any time for any reason without any consequences. The investigator can decide to let a subject out from the study for particular medical reasons, for example, serious adverse events. For every subject who decides to withdraw from the study, the reasons for withdrawal should be recorded.

### Randomization

Central randomization based on interactive web response system (IWRS,Brightech Clinical Information Management System) is carried out by Peking University Clinical Research Institute, which is independent of the trial administration office. The allocation sequence is computer-generated 1:1 with dynamic randomization system and is stratified by tumor size and neck lymph node metastasis.

### Blinding

Allocation status cannot be blinded for the participants and investigators due to different treatment methods and the visibility of implanted I-125 seed in CT image. However, the primary outcome (response to treatment) will be evaluated by an independent assessment board. Further, all statistical analysis will be done by a statistician in Peking University Clinical Research Institute who is not affiliated with the trial.

### Intervention –implanationt of I-125 seed

All subjects who are assigned to the intervention group will receive I-125 seed permanent implantation in the study. The operation of I-125 seed implantation will be conducted at the department of Oral and Maxillofacial Surgery, Peking University School and Hospital of Stomatology. I-125 seeds will be ordered from the manufacturer 1–2 days before surgical operation. The placement of the I-125 seed was determined from CT scans with the use of a brachytherapy treatment planning system (BTPS; Beijing Atom and High Technique Industries, Beijing, China). The I-125 seed (model 6711; Jaco Pharmaceuticals Co. Ltd., Zhejiang, China) activity was 0.9-1.0U per seed and had a half-life of 59.6 days. Clinical target volume (CTV) is defined as gross tumor volume (GTV) and its surrounding potential sub-clinical focus or microscopic focus, and it is also divided as CTV1 and CTV2. CTV2, namely high risk area, is defined as primary tumor and around 10 mm, as well as drainage regions of involved lymph node. CTV2, namely low risk area, is defined at 5 to 10 mm beyond CTV2 and lymphatic drainage area which should be prevented by irradiation. The prescribed dose is 120 Gy for CTV1 and 140 Gy for CTV2. And it is commonly assumed that irradiation dose can accumulate to prescribed dose in 6 months.

A CT scan is obtained one week after seeds implantation. The CT images in combination with BTPS are used to detect the location, number and isodose plot distribution of seeds. Re-implantation can be considered if implant related deficiencies are identified, including asymmetrical distribution, shedding or movement.

### Control group – IMRT

All subjects who are assigned to the control group will receive IMRT in the study, which is done at the department of radiotherapy, Peking University Cancer Hospital & Institute. Prescribed dose for planning target volume and dose segmentation is computed with simultaneous integrated boost modulated radiation therapy. In each fraction of irradiation treatment, a specific dose is used for different target volume during the entire course of treatment. Totally, 70 Gy IMRT in 33 fractions (5 fractions per week) are prescribed to the GTV, 60 Gy/33 fractions to CTV2, and 56 Gy/33 fractions to CTV1.

### Initial screening, assessment and follow-up

**Fig. 2:**
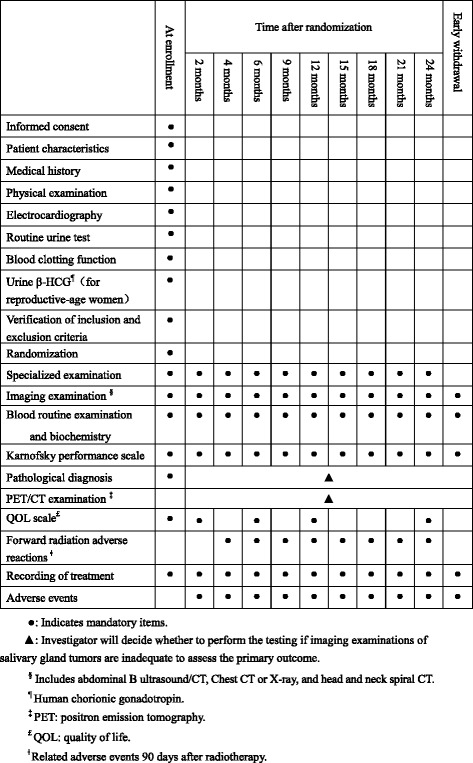
Observation, assessment, and follow-up schedule

After providing informed consent, potential participants will be asked standardized questions about their demographic characteristics and medical history. In addition, physical examination, electrocardiography, routine urine test, blood clotting function, urine β-human chorionic gonadotropin (HCG) for reproductive-age women, specialized examination, imaging examination, blood routine examination and biochemistry, pathological diagnosis and Karnofsky performance scale etc. will also be done and used for checking inclusion/exclusion criteria (Fig. 2).

After randomization, eligible participants will receive their treatment planning investigations and are followed-up following the same schedule for both intervention group and control group (Fig. 2) until recurrence, other malignancy or death is confirmed. Treatment duration is approximately 6–7 weeks for IMRT group. All the participants in both groups will be followed up at 2, 4, 6, 9, 12, 15, 18, 21 and 24 months after randomization, with specialized examination, imaging examination, blood routine examination and biochemistry, Karnofsky performance scale, recording of treatment and adverse events assessment done each time. Quality of life (QOL) scale will be assessed at 2, 6, 12, and 24 months. Forward radiation adverse reactions will be evaluated at each follow-up appointment except at 2 months after randomization. In addition, pathological diagnosis and PET/CT examination will be considered by the investigator if imaging examinations are inadequate to assess the primary outcome. Radioactive adverse events were classified by The National Cancer Institute-Common Terminology Criteria for Adverse Events (NCI-CTCAE) 4.0 standard. Chronic radioactive damages were classified by the criteria of Radiation Therapy Oncology Group/European Organization for Research and Treatment of Cancer (RTOG/EORTC), with common signs and severity recorded.

### Outcomes

Assessment of efficacy will be carried out by evaluation of imaging examination at each follow-up. If applicable, response to treatment will be evaluated according to the RECIST criteria (version 1.1) and classified as complete response (CR), partial response (PR), stable disease (SD) and progressive disease (PD). 

The primary outcome is estimated as the difference between the intervention and the control group in local control rate in 1 year. Local control rate is judged to have been attained if there is no evidence of PD at the primary site based on imaging findings and clinical examination at follow-up. However, distant metastasis and second primary cancer are not belonged to PD at the primary site in accordance with RECIST criteria (version 1.1) [28].

Secondary outcomes are a) progression-free survival (PFS), defined as the time from randomization to the earliest occurrence of PD in whole body or death due to any cause; PD in whole body includes PD at the primary site, distant metastasis and second primary cancer; b) overall survival (OS), from the date of randomization to the date of death from any cause or last date when the participant is alive; c) QOL evaluated using the European Organization for Research and Treatment of Cancer QOL Questionnaire (EORTC QLQ-C30 and QLQ-H&N35) of Chinese version; and d) safety of treatment.

### Sample size estimation

Based on medical record of subjects with inoperable salivary gland malignancies in Peking University Cancer Hospital & Institute and in Peking University School and Hospital of Stomatology, local control rate in 1 year was 81 % among subjects with I-125 PBT, and 45 % among subjects with IMRT. Based on a difference of 36 % between groups on the primary outcome, a total of 72 participants are required to provide 90 % power, with the use of two-sided significance level of 0.05. Assuming a 20 % drop-out rate, approximately 90 subjects (or 45 subjects per arm) will be enrolled.

### Statistical analysis

Analyses will be made using SAS statistical software (version 9.3, SAS Institute, Cary, NC, USA) by researchers at the Peking University Clinical Research Institute. The primary analyses will be done on an intent-to-treat basis and the last observation carry forward (LOCF) is used for missing values. Descriptive statistics will be used to summarize demographic and clinical characteristics of subjects randomised to the intervention and control group. The difference between two groups on demographic and clinical characteristics, response rate and drop-out rate will be compared using t-tests (or Wilcoxon rank sum test) and chi-square test/Fisher’s exact test as appropriate.

Primary outcome (local control rate in 1 year) analyses will be carried out using chi-squared tests. The survival curves (PFS and OS) are estimated by the Kaplan-Meier method, and log-rank test is used to test the null hypothesis that the respective curves are equal between the two groups. A two-sided significance level of 5 % is used. A covariance model will be used to compare the QOL between two groups by adjusting the difference of baseline. The incidence of adverse events between two groups is compared with the chi-square test/Fisher’s exact test.

## Discussion

This trail is conducted to prospectively evaluate I-125 PBT for inoperable salivary gland malignancies in terms of the efficacy, safety and the QOL.

Few studies have investigated the effect of I-125 PBT for inoperable salivary gland malignancies and thus, the evidence regarding I-125 PBT for inoperable salivary gland malignancies is sparse. In recent years, we have been exploring the effectiveness and feasibility of I-125 PBT for inoperable salivary gland malignancies. Our previous studies displayed that I-125 PBT may be one of the most promising treatment for inoperable salivary gland malignancies, but the evidence of these results is not sufficient enough due to limited number of observational studies [23, 24]. The proposed study is a randomised controlled single-centre trial conducted among subjects with inoperable salivary gland malignancies. To our knowledge, it is the first trial to investigate the efficacy of I-125 PBT for subjects with inoperable salivary gland malignancies, and will add to the knowledge base in a number of ways. Not only tumor cells, but also normal cells, are simultaneously killed by radiotherapy. Many side effects are often observed during and after radiotherapy, including lower white blood cell count, general weakness and loss of appetite, mucositis, xerostomia, hearing loss, radiation dermatitis, fibrosis, osteoradionecrosis of the mandible, and injury to optic apparatus. These side effects may lower the QOL of these subjects. In addition to effectiveness, we also investigate safety and QOL. These findings will help to systematically evaluate clinical application value of I-125 PBT.

We set a comparison treatment as the control arm, which will help us to assess the efficacy of I-125 PBT. We choose IMRT as the control arm because of its fewer side effects. IMRT is more precise radiotherapy modality which helps to reduce normal tissue damages compared with conventional external radiotherapy. Although there was no evidence that IMRT could significantly improve the LCR, many studies had shown that IMRT could significantly reduce toxic side effects when used in the head and neck cancer region [29]. This IMRT comparison arm can help to protect patient and improve compliance.

On account of the low prevalence, we will recruit patient with relatively inclusive entry criteria, and anticipate recruiting subjects across the spectrum of histologies and from various inoperable circumstances. These features will improve the generalisability of our findings. On the other hand, subjects will constitute a heterogeneous group, and this could weaken the power of our trial. We have taken a number of steps to reduce selection bias. The trial does not include all relevant subjects (e.g., some subjects are excluded, such as with subtotal resection, T1/T2-staged, and metastatic cancer). In addition, subjects are stratified by tumor size (≤4 cm vs. >4 cm) and neck lymph node metastasis (yes vs. no) to balance random allocation.

The treatment allocation cannot be blinded for participants and study staffs due to different procedures and the visibility of implanted I-125 seed in CT image, which is of course a limitation. However, to reduce observer bias in assessment, all assessment data are collected by research assistants who did not participate in the study; in addition, the primary outcome (response to treatment) will be evaluated by independent assessment board. Further, all statistical analysis will be done by a statistician at Peking University Clinical Research Institute who is not affiliated with the trial.

## Conclusion

The proposed study aims to investigate whether subjects with inoperable salivary gland malignancies will benefit from iodine-125 seed permanent brachytherapy. We will also study the side effects of such treatment.
